# The Role of Fall Rate From Transdermal Alcohol Concentration on Alcohol‐Related Consequences in College Students

**DOI:** 10.1111/acer.70369

**Published:** 2026-07-09

**Authors:** Yiping Li, Veronica L. Richards, Shannon D. Glenn, Robert J. Turrisi, Kimberly A. Mallett, Michael A. Russell

**Affiliations:** ^1^ Department of Biobehavioral Health The Pennsylvania State University University Park Pennsylvania USA; ^2^ TSET Health Promotion Research Center, Stephenson Cancer Center University of Oklahoma Health Sciences Tulsa Oklahoma USA; ^3^ Department of Health Promotion Sciences University of Oklahoma Health Sciences Tulsa Oklahoma USA; ^4^ Edna Bennett Pierce Prevention Research Center The Pennsylvania State University University Park Pennsylvania USA

**Keywords:** alcohol elimination rate, alcohol‐related consequences, self‐reported drinking, transdermal alcohol concentration (TAC) sensors, young adults

## Abstract

**Background:**

Alcohol elimination rate is associated with acute alcohol consequences, yet it remains difficult to measure reliably in real‐world settings. Wearable transdermal alcohol concentration (TAC) sensors provide a feasible option through passive, continuous monitoring of biological alcohol exposure. This facilitates precise testing of whether alcohol elimination rate predicts alcohol‐related consequences in naturalistic environments. Additionally, a faster alcohol elimination rate may buffer or reduce the risk of alcohol‐related consequences following high and/or rapid consumption.

**Method:**

Two observational studies (Alcohol Habits Study [*n* = 222] and Project ACE [*n* = 79]), each using a different alcohol sensor, were used in this study. Participants of both studies were young adults from universities who frequently engaged in heavy episodic drinking. Alcohol‐related consequences were collected through daily self‐reports the morning after drinking days and included metrics across multiple domains such as physical symptoms, interpersonal conflict, safety risk, sexual risk, and miscellaneous. Alcohol elimination rate and other dynamics were extracted from TAC‐positive trajectories for each day. Associations were tested using multilevel modeling.

**Results:**

Both studies showed that days with faster elimination rates were associated with more alcohol‐related consequences, although statistical significance was observed only in Project ACE. In the Alcohol Habits Study, a significant day‐level interaction indicated that the association between peak TAC and alcohol‐related consequences was reduced on days with faster alcohol elimination rates. Similar findings emerged in Project ACE but did not reach significance. Both studies demonstrated the same pattern of conditional association: as the daily fall rate increased, the simple association between peak TAC and alcohol‐related consequences diminished to the point of non‐significance.

**Conclusion:**

Our results provide novel evidence that alcohol elimination may have (a) an independent association with alcohol‐related consequences in natural settings and (b) a buffering impact on the association between peak TAC and alcohol‐related consequences. The results span two studies using two separate wearable sensors, supporting the validity of findings. Future research testing these associations in larger and more diverse samples is warranted.

## Graphical abstract

Using data from two observational studies (the Alcohol Habits Study [*n* = 222] and Project ACE [*n* = 79]), with different transdermal alcohol concentration (TAC) sensors, days with faster fall rates predicted higher frequencies of alcohol‐related consequences and moderated the association between days with peak TAC and alcohol‐related consequences.

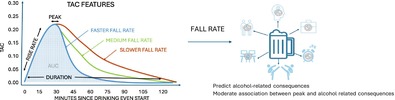

## 1 Introduction

Acute alcohol‐related consequences are a major health concern among young adults. They encompass a wide range of negative outcomes, such as blackouts, driving under the influence, and alcohol overdose that can occur during or after a drinking episode (D'Angelo et al. 2022; Hingson et al. 2017; Merrill et al. 2023; Richards et al. 2023). A wealth of research concludes that engaging in risky drinking behaviors puts young adults at increased risk of experiencing acute alcohol‐related consequences (D'Angelo et al. 2022; Merrill et al. 2023; Russell et al. 2022). Glenn et al. (2022) reported that college students who consumed alcohol experienced an average of 102 alcohol‐related consequences over a four‐year period in a longitudinal study. Identifying factors that predict alcohol‐related consequences is essential for reducing the acute harm of drinking. Prior research has shown that higher intoxication rates, elevated alcohol intake, and extended alcohol exposure are associated with increased consequences using various methodologies (Glenn et al. 2022; Howe and Finn 2024; Richards et al. 2023). While these studies have indeed advanced our understanding of how different alcohol absorptive phase dynamics (i.e., alcohol peak, absorption rate [rise rate], and duration) uniquely contribute to alcohol‐related consequences (Richards et al. 2023; Russell et al. 2022, 2024), they only provide part of the story. Differences in rates of alcohol *elimination* also likely affect consequence risk. Variation in elimination rate could shape further consumption behaviors during an episode and affect susceptibility to adverse outcomes. However, compared to other dynamics, the elimination rate (fall rate) has not been well studied in natural environments. This is probably because measuring the entire cycle of alcohol elimination outside of the laboratory has historically been either infeasible or impossible (Roehrs and Roth 2001). Alcohol elimination is an enzymatic process that cannot be measured by simple observation, nor can it be assessed through participants' self‐reports (Cederbaum 2012; Mackus et al. 2020). Assessment of alcohol elimination through breathalyzer testing is possible, but not practical because a substantial portion of elimination takes place during sleep (Kim et al. 2025).

Transdermal alcohol concentration (TAC) sensors are wearable devices that estimate alcohol exposure by measuring ethanol vapor that diffuses through the skin via passive perspiration. They offer a feasible option because they passively and continuously capture alcohol absorption and elimination processes in the field during both wake and sleep hours (e.g., Russell et al. 2022). The fluctuations in sensor readings collected by TAC generally correspond to alcohol exposure and metabolism overtime. To date, only a small number of studies have included TAC sensors to capture temporal alcohol concentration dynamics (*TAC features*) in the field (Courtney et al. 2023; Didier et al. 2025; Russell et al. 2022). Most studies using TAC have focused on ascending phases of TAC curves because of their direct links to alcohol consumption behaviors (Courtney et al. 2023; Didier et al. 2025; Richards et al. 2023; Russell et al. 2026) but did not include alcohol elimination (but see Richards, Glenn, et al. 2024; Richards, Turrisi, and Russell 2024; Russell et al. 2022 and Russell et al. 2024 for exceptions). Advances in passive monitoring of alcohol dynamics with TAC sensors in natural settings create new opportunities to incorporate elimination rates into the prediction of alcohol‐related consequences. The current focus is on examining whether differences in the rate of alcohol elimination (the process by which the body metabolizes and clears alcohol) may be predictive of a person's consequence risk and buffer the effect of peak BAC on those consequences.

Hereafter, we make a distinction between the *elimination rate* and the *fall rate*. Elimination rate refers to the rate at which the physiological elimination of ethanol occurs. Fall rate refers to the measured rate of decreasing ethanol concentration through the skin or breath. While both are ultimately tied to the same process, fall rates are measures, whereas elimination rates are biological processes (Cederbaum 2012). We write following this distinction throughout.

### 1.1 Alcohol Elimination Rates Vary Both Between and Within Persons

Alcohol elimination rates differ between young adults due to interindividual variability in alcohol metabolism. These differences are determined and have shown associations with a number of factors. For example, adults with higher alcohol dehydrogenase (ADH) activity in the liver metabolize alcohol more efficiently and recover from intoxication more quickly than those with lower activity (Neumark et al. 2001; Zakhari 2006). The specific isoform of ADH also plays a role in alcohol metabolism; individuals expressing the beta‐3 class I ADH isoform exhibit faster alcohol elimination than those with the beta‐1 isoform (Cederbaum 2012; Thomasson et al. 1995). Considering the rate of biological processing, liver damage can impair ADH function and expression, slowing the rate of alcohol elimination (Cederbaum 2012). Studies have shown that alcohol elimination rates differ by sex (Baraona et al. 2001; Cederbaum 2012) and race/ethnicity (Cederbaum 2012).

It is less widely recognized outside research settings that alcohol elimination rates can fluctuate from day to day within the same individual. These fluctuations result from an interplay of behavioral and physiological factors influencing metabolic function, including the interplay between alcohol elimination rate and consumption volume. When BAC is at a higher concentration, the alcohol metabolism rate is faster than at lower concentrations (Cederbaum 2012; Jones 2019; Simic and Tasic 2007). The metabolism rate is approximately 50% higher at 83 μmol/min per liter (Keiding et al. 1983). Concordant with these results, previous research using TAC sensors has observed that an individual's alcohol elimination rates can vary across drinking days (Russell et al. 2022). Dietary intake before alcohol consumption can also affect the alcohol metabolism rate. Carbohydrates, proteins, and fats can facilitate the conversion of NADH back to NAD^+^ and enhance oxygen utilization in the mitochondria (Cederbaum 2012). This process increases liver blood flow and accelerates the alcohol elimination rate (Cederbaum 2012). Compared to drinking in a fasted state, alcohol consumed on fed days is eliminated more quickly, resulting in lower peak blood alcohol concentrations and a shorter duration of intoxication (Cederbaum 2012; Ramchandani et al. 2001). Alcohol metabolism may be regulated by circadian rhythms and influenced by fluctuations in core body temperature throughout the day (Cederbaum 2012; Sturtevant and Garber 1988). A study demonstrates that alcohol elimination rates are higher during the evening and slower in the morning (Jones and Parades 1974). Other factors (e.g., exercise, medications, age, etc.) may also contribute to day‐to‐day variation in alcohol metabolism rate.

### 1.2 Alcohol Elimination and Associated Consequences

The documented evidence from laboratory studies shows that variations in alcohol elimination rates are associated with alcohol‐related consequences. Alcohol metabolism and excretion are the two primary components of alcohol elimination. Metabolism occurs in the liver, while excretion takes place through urine, sweat, and breath (Cederbaum 2012; Zakhari 2006). Elimination rate corresponds to differences in blood alcohol concentration (BAC). When BAC is low (below 0.19 g/kg) (Høiseth et al. 2016), alcohol elimination follows first‐order kinetics, meaning it increases in direct proportion to BAC (Simic and Tasic 2007; Wagner et al. 1985). A higher elimination rate during this phase reduces the extent of intoxication from rapid drinking before reaching the lower boundary of high BAC. When the BAC level surpasses approximately 0.15–0.20 g/L (Jones 2019), the association is disrupted because the alcohol elimination rate enters zero‐order kinetics and tends to remain constant (Simic and Tasic 2007; Wagner et al. 1985). Young adults with faster elimination rates exhibit steeper BAC fall rates, resulting in shorter durations of intoxication and reduced effects of alcohol impairment (alcohol elimination rate = $\frac{\text{Peak BrAC}\left(\%\right)}{\text{Time to reach BrAC}=0}$). One study measured elimination rate using breathalyzers (the Dräger Alcotest 7410Plus COM). After alcohol consumption, breathalyzer testing was conducted to repeatedly capture the BAC until participants reached a breath alcohol concentration (BrAC) of zero on two consecutive measurements (Mackus et al. 2020). Results showed that participants with faster elimination rates experienced less intense hangovers compared to individuals with slower elimination rates who were given the same amount of alcohol (Mackus et al. 2020). Another association may appear over longer spans of time: those with faster elimination, facilitating riskier and more prolonged drinking behaviors, might show an elevated risk for alcohol use disorder. In a lab‐controlled study, participants consumed two alcoholic beverages under supervision (Boyd and Corbin 2018). After the initial measurements were completed, participants were given the opportunity to freely consume beverages they believed to be alcoholic beer (Boyd and Corbin 2018). Social drinkers with a faster (vs. slower) alcohol metabolism rate tended to continue their drinking behaviors after peak BAC, increasing the potential risk for excessive alcohol consumption (Boyd and Corbin 2018). Continued drinking after peak BAC without further increases can occur when a person drinks slowly or at a low level. Individuals may not be consuming alcohol quickly enough to overcome their biological rate of alcohol metabolism. When this occurs, BAC may plateau or begin to decline even with continued consumption. These research findings highlight the importance of assessing alcohol elimination rate to better understand variability in alcohol‐related consequences.

### 1.3 Alcohol Elimination From TAC Readings

Alcohol elimination rate in natural environments can indirectly be inferred using wearable TAC sensors. TAC sensors capture the amount of ethanol that diffuses through the skin via sweat or vapor non‐invasively and continuously (Barnett et al. 2014; Russell et al. 2022). When drinking episodes begin, ethanol vapor can be detected by TAC sensors shortly afterward (approximately 30–45 min on average; Barnett et al. 2014; Clapp et al. 2017; Russell et al. 2022). Data collected by TAC sensors are visualized as a TAC curve (Russell et al. 2022; Simons et al. 2015). From this curve, alcohol intoxication “features,” including *peak*, *rise rate*, *fall rate*, and *duration*, are extracted and used to characterize drinking behavior and predict alcohol‐related outcomes (Roache et al. 2019; Russell et al. 2022). TAC peak corresponds to the peak level of alcohol exposure during the episode (Roache et al. 2019; Russell et al. 2022). TAC duration reflects the total time required for the body to absorb and eliminate alcohol (Roache et al. 2019; Russell et al. 2022). TAC rates (rise and fall rates) are calculated as the change in TAC divided by the change in time (Roache et al. 2019; Russell et al. 2022). The *rise rate* captures the speed of increasing alcohol concentration during the episode or day, while the *fall rate* captures the speed of decreasing alcohol concentration during the episode or day (Roache et al. 2019; Russell et al. 2022).

### 1.4 Current Study

While growing research appears to support that fall rate is linked to various types of alcohol‐related consequences, very little research has examined their association in observational studies. Fall rate derived from the TAC curve is an important biological marker that reflects the alcohol elimination rate. Differences in fall rate may be driven by (a) faster or slower alcohol metabolism after drinking has stopped and (b) continued low‐level drinking that does not raise TAC levels but prevents them from falling as fast as they would if drinking had stopped. There may therefore be untapped information in TAC fall rates that would aid in the prediction of alcohol‐related consequences. To examine the role of fall rate on alcohol‐related consequences, the current study tests the role of fall rate in day‐to‐day experiences of alcohol‐related consequences in young adults' natural settings. Since the alcohol elimination rate depends on total alcohol consumption (Cederbaum 2012; Simic and Tasic 2007), examining the role of peak is necessary for precise estimation of the association between fall rate and alcohol‐related consequences. The dynamic relationship between fall rate and peak also suggests that the effect of peak on alcohol‐related consequences may vary based on the fall rate. Using two studies of young adults who wore TAC sensors and completed daily surveys about their alcohol use: Alcohol Habits Study (*n* = 222) and Project ACE (*n* = 79), we tested the following hypotheses separately in each study to allow cross‐validation:

*Days with higher peaks and slower fall rates (adjusted for peak) will be associated with more alcohol‐related consequences compared to days with lower peaks and faster fall rates*.

*People with higher average peaks and slower average fall rates will experience more alcohol‐related consequences compared to people with lower average peaks and faster average fall rates*.

*Buffering: The day‐level association between peak and alcohol‐related consequences will be moderated by the day‐level fall rate and the person's average fall rate. Those with faster average fall rates will show a weaker association between day‐level peaks and alcohol‐related consequences, and days with faster fall rates will be associated with weaker relationships between TAC peaks and alcohol‐related consequences*.

## 2 Method

### 2.1 Study 1 Method

#### 2.1.1 Participants and Procedures

Participants in Study 1 (The Alcohol Habits Study) included 222 young adults (*M* age = 22.3 years, 64% female, 79% non‐Hispanic white; 84% undergraduate) recruited near the campus of a large northeastern US university (Russell et al. 2022). The analyses of the current report and the study design were not preregistered. Given the lack of relevant previous effect sizes on which to base power calculations, we did not conduct a priori power analyses for these data. Sample size was determined using guidelines for intensive longitudinal data (Bolger and Laurenceau 2013). Participants completed a screening survey prior to enrollment. For eligibility, participants needed to: (1) be between the ages of 21–29, (2) have engaged in heavy episodic drinking (HED) at least weekly on average during either the past calendar year or typically during the academic year, and (3) be sufficiently proficient in written English to complete study procedures. HED was defined as consuming 4+/5+ drinks in a row for females/males (Wechsler et al. 1995). A standard drink was defined for participants as “a glass of wine, a bottle of beer, a wine cooler, or a shot glass of liquor on its own or mixed.” Participants were not screened for psychiatric disorders. Five hundred and thirty‐one completed the screening survey, and 419 were eligible. Invitations were sent on a “first‐come, first serve” basis according to the order in which screening surveys were received. Time and resource limitations prevented invitations to all eligible participants, leading us to invite 343 individuals to participate. Of the 343 invited, 222 completed the study. No evidence of bias was observed comparing those completing versus not completing by gender, race/ethnicity, student status, or past‐two‐week binge drinking (*p*s > 0.10).

The study consisted of five 24‐h periods spanning six consecutive days. All participants began the study on a Wednesday and finished on a Monday, thus capturing the “social weekend” of Thursday, Friday, and Saturday in which most drinking tends to occur (Finlay et al. 2012). Data collection took place across 25 weeks from November 2017 to November 2018 and across 8 weeks from November 2019 to March 2020. The study included baseline and endpoint assessments, three times daily ecological momentary assessment (EMA), participant‐initiated drinking‐episodic EMA, and transdermal sensors. All procedures were approved by the university's institutional review board. Data and analytic code are not publicly available but will be made available (in accordance with IRB standards) upon request from the first author. We report all data exclusions, manipulations, and measures in the study.

##### 2.1.1.1 EMA Protocol

Participants were provided with an Android‐based smartphone. The device had a custom application (“app”) designed by specialist EMA survey programmers at the study investigator's university. The app contained two survey types. First was a three‐times daily scheduled EMA report, which prompted participants to complete surveys at fixed times: morning (10 a.m.), afternoon (4 p.m.), and evening (9 p.m.). Participants could choose to have these prompts occur 1 h later (11 a.m., 5 p.m., 10 p.m.; 26% chose this option). Participants could respond immediately upon being prompted or could self‐initiate surveys prior to or after the prompt, depending on their schedule that day. Daily reports of drinking behavior were collected in the morning diary, referring to “yesterday's/last night's drinking.” Compliance was high; 94% of scheduled morning reports were completed, and 94% of these were initiated within 2 h of the prompt time (median absolute time difference = 26.5 min; IQR = 11.3, 50.4 min). Morning surveys were completed within 3.9 min (IQR = 2.5, 4.3) of starting.

The second survey type was the episodic EMA, which consisted of a participant‐initiated survey sequence with timed follow‐ups occurring every 30 min during ongoing drinking events. The current analyses do not use these episodic EMA data; this information is presented here only to provide the full context of the study.

##### 2.1.1.2 TAC Sensor Protocol

Participants wore the SCRAM‐CAM anklet during wake and sleep hours. After data collection, SCRAM‐CAM data are uploaded to the company's online server (SCRAMNet), which houses TAC data, records TAC “positives,” and tracks compliance with device wear through skin temperature and sensor quality (infrared voltage) readings. Compliance rates were high. Only 2.0% of TAC data showed evidence of device removal or interference. These data points were clustered within a minority of individuals (*n* = 24). No evidence suggested that compliance was associated with study demographics (gender, age, race/ethnicity, student status) or AUDIT scores (*p*s > 0.05). We began with 52,726 TAC observations collected from 218 individuals; data from 4 participants were lost due to device failure. Drinking episodes were then identified and coded using validated research guidelines informed by controlled administration studies (Roache et al. 2019). These guidelines (a) increase the sensitivity of detection for low‐ to moderate‐level drinking with little sacrifice of specificity and (b) remove events and observations that indicate environmental alcohol contamination (e.g., spilled drinks near the device, fumes from cleaning products; Roache et al. 2019). Details of initial TAC data processing are offered in Russell et al. (2022). Following episode identification and exclusions, we retained 608 TAC drinking events containing 16,385 data points among 195 participants (87.8% of the sample). Prior to analysis, TAC data were smoothed to remove noise and facilitate feature extraction using penalized b‐splines (Eilers and Marx 1996). The number of drinks per episode was recorded only in Study 1. The mean number of standard drinks consumed during EMA‐measured drinking episodes was 6.62, with a median of 5.5. The distribution of drinks per episode suggests that approximately 54% of episodes were classified as heavy drinking events.

### 2.2 Study 2 Method

#### 2.2.1 Participants and Procedures

Study 2 consisted of seventy‐nine participants aged 18–22 who regularly engaged in HED (55.7% female, 86.08% non‐Hispanic White; 50.63% Junior) (Richards, Glenn, et al. 2024; Richards, Turrisi, and Russell 2024). Sample size was determined using Monte Carlo power simulations in Mplus, which showed that a sample size of 80 participants, each providing 12 days of data, provided over 95% power to detect effect sizes of 0.2 SD or greater. We randomly selected 6000 students (50% sophomores and 50% juniors based on credits) from the registrar's database at a large public university in the northeastern US. Participants received an email describing the study and inviting participation. Emails included personalized URLs for the screening survey. Students were eligible to participate if they were aged 18–22 years, in their second or third years of college, reported drinking > four drinks on a typical Friday or Saturday in the past semester, experienced at least one alcohol‐induced blackout in the past semester, owned an iPhone (necessary for the BACtrack Skyn software), and were willing to wear a sensor each social weekend (Thursday night through Sunday morning) for four consecutive weekends. Second‐ and third‐year students were recruited because this is a developmentally important time for alcohol risk (e.g., moving off campus, increased autonomy, transitioning into legal drinking age). Eligible participants were immediately redirected to the baseline survey. Following the baseline survey, students scheduled their in‐person enrollment visit. Enrollment visits took place Monday through Thursday prior to the start of the study's ambulatory assessments. All procedures were approved by the university's Institutional Review Board. The study was not preregistered. We report how we determined our sample size, all data exclusions, all manipulations, and all measures in the study. Data are available upon request from the first author.

At screening, 15.5% (*n* = 927) consented to participate; 73.1% of those who consented (*n* = 678) completed the screening. 28.3% (*n* = 192) of screened participants were eligible to continue, and 100% of eligible students completed the baseline survey. Due to the BACtrack Skyn device availability, only 80 students were invited to attend an enrollment visit. Prior to enrollment visits, one dropped from the study, resulting in a final sample of 79.

##### 2.2.1.1 Daily Diary and Sensor Protocol

We used the BACtrack Skyn model T15 sensor. Students were instructed to wear sensors for four consecutive weekends, starting Thursday at 5 p.m. and continuing until Sunday morning after waking. They received an email and a text message at 4 p.m. each Thursday reminding them to turn on and wear the device by 5 p.m. Students were instructed to charge devices once a week, prior to each weekend. Surveys were sent to all students each weekend morning (Friday–Sunday) at 10 a.m., covering the day/night before. Surveys were available until 6 p.m. each day. To enhance compliance and retention, students received an email and text reminder on Monday prior to the start of each social weekend. An email reminder to complete the survey at 1 p.m. and a text message reminder at 4 p.m. each weekend day were also sent to students. Survey compliance was 89.9%. Participants were compensated $15 for completing the baseline survey and an additional $5 for each survey completed (up to $75 total). Once participants completed 9 surveys, they were entered in a raffle to win 1 of 8 $100 gift cards.

#### 2.2.2 Day‐Level TAC Data

##### 2.2.2.1 Initial Processing

The sensor assessed TAC every 20 s, resulting in 443,999 observations across our 79 participants. Drinking episodes were coded using published guidelines (Courtney et al. 2023; Didier et al. 2025; Richards et al. 2022). We applied algorithms to filter out observations in which the sensor was turned on but not worn (Didier et al. 2025). These algorithms are based on temperature and movement sensors. First, if skin temperature was greater than 28°C, the sensor was considered worn (88% of observations). Second, if the temperature was less than or equal to 28°C but more than 5°C above the participant's minimum, it was considered worn (an additional 7.4% of observations). Third, if the temperature was more than 3°C above the participant's minimum and the motion sensor registered above 0.01 Gs, it was considered worn (an additional 0.4% of observations).

##### 2.2.2.2 Identifying Drinking Episodes

Data were then smoothed using a 30‐min centered moving average. Following manufacturer instructions, negative TAC values were recoded to 0 after smoothing. The start of an episode was indicated if (a) there were two consecutive 0 readings followed by at least one positive (TAC > 5) reading, and (b) there was a positive at the start of the data stream and at least one positive on the next two measurement occasions. The end of an episode was indicated if (a) an alcohol‐positive observation was followed by two consecutive non‐positive readings, (b) the last alcohol‐positive observation was the last reading for the person, or (c) an alcohol‐positive observation was followed by a negative reading that was the person's last observation. Episodes were divided into two if consecutive observations were more than 30 min apart. False positive episodes were filtered out using published guidelines (Courtney et al. 2023; Didier et al. 2025; Richards et al. 2022). Our specific criteria focused on identifying TAC curves with features that were biologically implausible according to research and manufacturer information. We removed episodes that (1) were less than 45 min in duration, (2) were less than or equal to 60 min in duration and had a peak greater than or equal to 400 μg/L air, and/or (3) had rise and fall rates that did not fall between +/− 20 and 300. This removed 38% of the originally identified episodes, but these episodes contained only 6.8% of the total sensor data, leaving 718 drinking episodes with valid TAC data. Commensurate with previous studies (Courtney et al. 2023; Richards et al. 2022; Russell et al. 2022), TAC sensors captured 77% of self‐reported drinking days.

### 2.3 Social Days

Drinking behavior does not conform to the midnight‐to‐midnight boundaries of a calendar day, and retrospective morning reports of yesterday's/last night's drinking likely included hours after midnight. In both studies, day boundaries for TAC and EMA drinking episode data were redefined with 10 a.m. marking the start of a new “social” day. 10 a.m. was chosen because it was the modal prompt time for the morning report, which asked participants to reflect on their drinking the day/night before. If participants had any valid TAC data on a social day, it was considered a drinking day.

### 2.4 Measures

TAC features peak and fall rates were extracted from each social day with TAC‐positive episode data in both studies. Peak TAC was the maximum TAC value for each social day. Fall rate was the average rate of all descending point‐to‐point TAC rates for that social day. For days with no TAC‐positive events, all features were coded as 0 if (a) no evidence of non‐compliance s(removal or interference) of the SCRAM sensor was observed that day in Study 1; or (b) the BACTrack sensor was worn for 80% or more of the hours of the social day, but there were no episodes present in Study 2. Study 1 contained 1274 days of TAC data across 218 persons for analysis; Study 2 contained 718 days of TAC data across 78 persons for analysis.

#### 2.4.1 Alcohol‐Related Consequences

In both studies, 13 negative alcohol‐related consequences were assessed in the morning diary following self‐reported drinking days only. Consequences were selected from the Importance of Consequences of Drinking Short Form (Patrick and Maggs 2011) and the Brief Young Adult Alcohol Consequences Questionnaire (adapted for daily use; Kahler et al. 2005). The specific consequences chosen were intended to provide a comprehensive listing across multiple domains, including physical symptoms (e.g., hangover, sick to your stomach, throw up), interpersonal conflict (e.g., get into an argument, get into a physical fight), safety risk (e.g., blackout, wake up in an unexpected place), sexual risk (e.g., have a sexual experience you wish you had not), and miscellaneous (get into trouble with the police or campus authorities, find yourself in a situation where no one was sober enough to drive). As in previous studies (Richards, Glenn, et al. 2024; Richards, Turrisi, and Russell 2024; Richards et al. 2025; Russell et al. 2022, 2024), alcohol‐related consequences were “led” 1 day (shifted up one row in the daily data file) prior to analysis to align day‐level retrospective reports with prospectively collected TAC features.

### 2.5 Statistical Analysis

Means and day‐, weekend‐, and person‐level SDs for variables measured daily (TAC features and day‐level consequences) were generated from empty multilevel linear models in R. All hypotheses were tested using Bayesian multilevel modeling with non‐informative priors and 20,000 iterations (50% warmup) across two chains using the brms R package (Bürkner 2017). Bayesian models were used to facilitate convergence of random effects. Model estimates are the medians of the posterior parameter distributions. We use the term credible to describe associations that would be described as significant in a frequentist framework. To determine credibility, we used 95% credibility intervals (CrIs; Makowski et al. 2019). If the 95% CrI did not include 0, then the association was deemed credible. Study 1 had days nested within persons, so two‐level analyses were used. Study 2 had days nested within weekends and weekends nested within persons, so three‐level analyses were used for Study 2. Separate models were conducted for each study. Models used a negative binomial outcome distribution and reported incident rate ratios (IRR) to describe associations. Cluster‐level means of TAC features (person‐means in Study 1, person‐ and weekend‐level means in Study 2) were included in all models. This partitioned the variance in TAC features and facilitated interpretation of TAC feature‐consequence regression coefficients as day‐level associations, comparing days within the same persons and/or person‐weekends. This within‐person, within‐weekend comparison naturally adjusts for all confounding variables that are stable at these levels, whether measured or unmeasured, thereby removing the need to statistically control for variables at these levels (Allison 2005; Hamaker and Muthén 2020; Hoffman and Stawski 2009). All TAC features were z‐scored to allow for meaningful comparison of their effect sizes. Each model included random intercepts for both the person‐ and weekend‐ levels (weekend‐level intercepts included in Study 2 only). Day‐level random slopes were included in models testing Hypothesis 2, which allowed day‐level associations to vary between persons. This allowed us to test moderation of day‐level peak TAC‐consequence associations by person‐mean fall rate while avoiding the assumption that all person‐level variability in the peak‐consequence slope was explained by the moderator. Linear combinations representing simple slopes and estimated consequence levels, along with their 95% credible intervals, were generated from moderation models using posterior distributions.

## 3 Results

### 3.1 Descriptive Results

Demographic information from two studies is presented in Tables 1 and 2, respectively. In Study 1, the final sample consisted of 222 young adults (63.5% female, 36.5% male), with a mean age of 22.3 years (SD = 1.34) and a mean bodyweight of 157.7 pounds (SD = 33.50). The majority of participants identified as non‐Hispanic White (78.8%). In Study 2, the final sample consisted of 79 undergraduate students (55.7% female, 44.3% male), with a mean age of 20.06 years (SD = 0.91) and a mean bodyweight of 155.83 pounds (SD = 28.97). The majority of participants identified as White (86.1%) and non‐Hispanic/Latino (88.6%).

**TABLE 1:** Participant demographics of Study 1.

	***N***	**%**
Gender		
Female	141	63.50
Male	81	36.50
	**M**	**SD**
Age	22.3	1.34
Bodyweight (in pounds)	157.7	33.50
	***N***	**%**
Race, non‐Hispanic ethnicity		
White	175	78.80
Asian	15	6.80
Black	8	3.60
Native American	0	0.00
Mixed	8	3.60
Race, Hispanic ethnicity		
White	11	5.00
Asian	0	0.00
Black	0	0.00
Native American	1	0.50
Mixed	3	1.40
Student status		
Undergraduate student	187	84.20
Graduate, professional, or other student	14	6.40
Nonstudent	21	9.50
Typical heavy drinking frequency during the academic year		
1 day per week	87	39.4%
2 days per week	71	32.1%
3 days per week	54	24.4%
4 or more days per week	9	4.1%
Heavy drinking episode in the past 2‐weeks		
0 times	5	2.3%
1 time	34	15.3%
2 times	56	25.2%
3–5 times	89	40.1%
6–9 times	31	14.0%
10 or more times	7	3.2%

**TABLE 2:** Participant demographics of Study 2.

	***N***	**%**
Gender		
Female	44	55.70
Male	35	44.30
	**M**	**SD**
Age	20.06	0.91
Bodyweight (in pounds)	155.83	28.97
	***N***	**%**
Race		
Asian	4	5.06
Black	1	1.32
Multiracial	3	3.09
White	68	86.08
Other	3	3.80
Ethnicity		
Hispanic/Latino	9	11.39
Not Hispanic/Latino	70	88.61
Year in school		
Sophomore	39	49.37
Junior	40	50.63

### 3.2 Model Results

#### 3.2.1 Hypothesis 1

Hypothesis 1 stated that days with higher peaks and slower fall rates (adjusted for peak) would be associated with more alcohol‐related consequences compared to days with lower peaks and faster fall rates across two studies. Results are presented in Tables 3 and 4. In Study 1, we found no evidence that daily fall rate was associated with alcohol‐related consequences. However, the daily peak was a significant predictor of alcohol‐related consequences. Specifically, on days when participants experienced a peak TAC that was one standard deviation above their average peak, the number of alcohol‐related consequences experienced was about two times higher than on days when their peak TAC was at their own average (IRR = 2.01, 95% CrI: 1.55, 2.47).

**TABLE 3:** Regression results predicting the alcohol related consequences of Study 1.

Model 1			
Fixed effects	**IRR**	**CrI_low**	**CrI_high**
Intercept	0.16	0.11	0.22
Daily fall rate	0.89	0.71	1.11
Daily peak	**2.01**	**1.55**	**2.47**
Person‐mean fall rate	1.05	0.85	1.30
Person‐mean peak	**1.40**	**1.08**	**1.78**
Female (ref: Male)	1.28	0.94	1.64
Weight	0.92	0.68	1.22
Weekday (ref: Weekend)	1.03	0.82	1.25
Random effects	**Estimates**	**CrI_low**	**CrI_high**
SD of random intercept	0.92	0.64	1.21
Model 2			
Fixed effects	**IRR**	**CrI_low**	**CrI_high**
Intercept	0.13	0.08	0.19
Daily fall rate	0.96	0.72	1.26
Daily peak	**2.06**	**1.60**	**2.62**
Person‐mean fall rate	1.04	0.85	1.27
Person‐mean peak	**1.39**	**1.06**	**1.77**
Female (ref: Male)	1.23	0.90	1.59
Weight	0.89	0.65	1.16
Weekday (ref: Weekend)	1.06	0.84	1.29
Random effects	**Estimates**	**CrI_low**	**CrI_high**
SD of random intercept	1.18	0.83	1.53
SD of the random slope of daily fall rate	0.41	0.05	0.69
SD of the random slope of daily peak	0.13	0.00	0.39
Correlation between random intercept and random slope of daily fall rate	−0.68	−1.00	−0.05
Correlation between random intercept and random slope of daily peak	−0.15	−0.96	0.73
Correlation between the random slope of daily fall rate and the random slope of daily peak	−0.30	−0.97	0.71
Model 3			
Fixed effects	**IRR**	**CrI_low**	**CrI_high**
Intercept	0.09	0.05	0.14
Daily fall rate	**1.76**	**1.13**	**2.55**
Daily peak	**2.83**	**2.08**	**3.81**
Person‐mean fall rate	1.14	0.83	1.52
Person‐mean peak	**1.37**	**1.04**	**1.73**
Interaction: Daily peak X Daily fall rate	**0.70**	**0.58**	**0.83**
Interaction: Daily peak X Person‐mean fall rate	0.93	0.81	1.08
Female (ref: Male)	1.26	0.95	1.61
Weight	0.89	0.65	1.15
Weekday (ref: Weekend)	1.05	0.85	1.26
Random effects	**Estimates**	**CrI_low**	**CrI_high**
SD of random intercept	1.42	1.02	1.80
SD of the random slope of daily fall rate	0.59	0.29	0.87
SD of the random slope of daily peak	0.16	0.00	0.42
Correlation between random intercept and random slope of daily fall rate	−0.87	−1.00	−0.55
Correlation between random intercept and random slope of daily peak	−0.23	−0.93	0.71
Correlation between the random slope of daily fall rate and the random slope of daily peak	−0.09	−0.89	0.83

*Note:* All variables are centered and standardized. Significant fixed effects (CrIs that do not contain 0.0) are bolded.

**TABLE 4:** Regression results predicting the alcohol related consequences of Study 2.

Model 1			
Fixed effects	**IRR**	**CrI_low**	**CrI_high**
Intercept	0.59	0.45	0.74
Daily fall rate	**1.25**	**1.05**	**1.46**
Week‐mean fall rate	1.55	0.98	2.27
Person‐mean fall rate	1.57	0.72	2.73
Daily peak	**1.22**	**1.02**	**1.45**
Week‐mean peak	1.31	0.79	1.99
Person‐mean peak	0.86	0.44	1.39
Female	0.80	0.61	0.97
Weights	1.13	0.89	1.43
Random effects	**Estimates**	**CrI_low**	**CrI_high**
SD of random intercept	0.55	0.31	0.82
SD of random intercept at the week	0.19	0.00	0.52
Model 2			
Fixed effects	**IRR**	**CrI_low**	**CrI_high**
Intercept	0.57	0.43	0.71
Daily fall rate	**1.24**	**1.01**	**1.45**
Week‐mean fall rate	**1.55**	**1.05**	**2.31**
Person‐mean fall rate	1.58	0.80	2.85
Daily peak	**1.26**	**1.05**	**1.57**
Week‐mean peak	1.31	0.79	1.93
Person‐mean peak	0.81	0.42	1.34
Female	0.79	0.62	0.98
Weights	1.13	0.89	1.42
Random effects	**Estimates**	**CrI_low**	**CrI_high**
SD of random intercept	0.60	0.35	0.91
SD of the random slope of fall rate	0.12	0.00	0.34
SD of the random slope of peak	0.18	0.00	0.38
SD of random intercept at the week	0.20	0.00	0.49
Correlation between the random intercept and the random slope of daily fall rate	0.02	−0.84	0.87
Correlation between the random intercept and the random slope of daily peak	−0.59	−0.99	0.34
Correlation between the random slope of daily fall rate and the random slope of daily peak	−0.23	−0.97	0.74
Model 3			
Fixed effects	**IRR**	**CrI_low**	**CrI_high**
Intercept	0.56	0.42	0.71
Daily fall rate	**1.33**	**1.09**	**1.65**
Week‐mean fall rate	1.56	0.95	2.24
Person‐mean fall rate	1.93	0.79	3.44
Daily peak	**1.41**	**1.10**	**1.78**
Week‐mean peak	1.33	0.83	2.00
Person‐mean peak	0.75	0.36	1.21
Interaction: daily peak X daily fall rate	0.90	0.79	1.02
Interaction: daily peak X person‐mean fall rate	0.86	0.58	1.16
Female	0.81	0.64	1.00
Weights	1.13	0.88	1.41
Random effects	**Estimates**	**CrI_low**	**CrI_high**
SD of random intercept	0.60	0.29	0.91
SD of the random slope of fall rate	0.17	0.00	0.45
SD of the random slope of peak	0.23	0.00	0.46
SD of random intercept at the week	0.23	0.00	0.56
Correlation between the random intercept and the random slope of daily fall rate	−0.03	−0.71	0.83
Correlation between the random intercept and the random slope of daily peak	−0.52	−0.98	0.23
Correlation between the random slope of daily fall rate and the random slope of daily peak	−0.06	−0.88	0.69

*Note:* All variables are centered and standardized. Significant fixed effects (CrIs that do not contain 0.0) are bolded.

In Study 2, both daily fall rate and daily peak were significant unique predictors of alcohol‐related consequences. Within‐person associations suggested that on days when the daily fall rate or peak was one standard deviation higher than the individual's weekly average, the number of alcohol‐related consequences increased by 25% and 22%, respectively (IRR_fall = 1.25, 95% CrI: 1.05, 1.46; IRR_peak = 1.22, 95% CrI: 1.02, 1.45). These associations remained significant after including random slopes.

#### 3.2.2 Hypothesis 2

Hypothesis 2 examined whether individuals with higher average peaks and slower average fall rates would experience more alcohol‐related consequences compared to individuals with lower average peaks and faster average fall rates. Results are presented in Tables 3 and 4. In Study 1, there was no association between‐person fall rate and alcohol‐related consequences. However, participants whose average peak TAC was one standard deviation higher than the sample average experienced 40% more alcohol‐related consequences on average (IRR = 1.40, 95% CrI: 1.08, 1.78). This result remained statistically significant after including random slopes for daily peak and daily fall rate, with only slight attenuation of the IRR. The standard deviation of the random intercept suggests that the likelihood of experiencing alcohol‐related consequences varied across participants on average.

#### 3.2.3 Hypothesis 3

Hypothesis 3 proposed that day‐level fall rate would moderate the day‐level association between peak and alcohol‐related consequences. Individuals with faster average fall rates were expected to show a weaker association between day‐level peaks and alcohol‐related consequences, and days with faster fall rates were expected to be associated with weaker relationships between TAC peaks and alcohol‐related consequences. We allowed the within‐person association between peak and alcohol‐related consequences to vary by both daily and person‐mean fall rate using interaction terms in both studies (Table 4).

**FIGURE 1:**
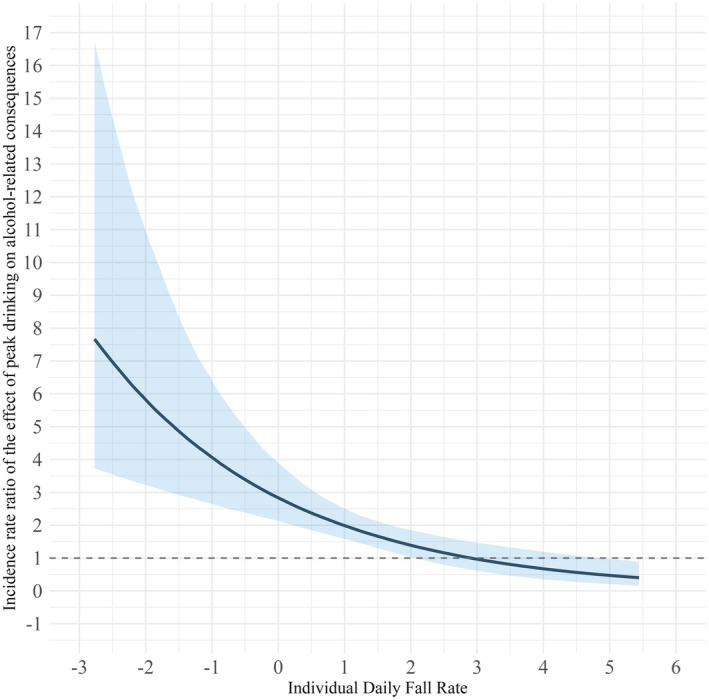
Within‐person association between peak and alcohol‐related consequences differed by daily fall rate (study one). The within‐person association between peak drinking and alcohol‐related consequences varied by daily fall rate, with the person‐mean fall rate held constant. The x‐axis represents individuals' daily fall rates (standard deviations above or below each participant's mean fall rate), and the y‐axis represents the incidence rate ratio (IRR) of the effect of peak drinking on alcohol‐related consequences. The gray shaded area indicates the credible interval.

In Study 1, models moderated by daily and person‐mean fall rate showed that daily peak was significantly associated with consequences at the lower range (from the 1st to the 59th percentiles) and higher range (from the 93rd to the 99th percentiles) of daily fall rate (Figure 1). The association between peak and alcohol‐related consequences was significantly reduced when the daily fall rate was between 2.04 and 4.86 standard deviations above the individual's average. The significant range of the within‐person interaction by daily fall rate extended from 2.77 standard deviations below to 2.04 standard deviations above participants' own average fall rate, as well as from 4.86 to 5.44 standard deviations above their average. The interaction effect indicated that the effect of daily peak on alcohol‐related consequences was 30% smaller for each one standard deviation increase in daily fall rate (IRR = 0.70, 95% CrI: 0.58, 0.83).

**FIGURE 2:**
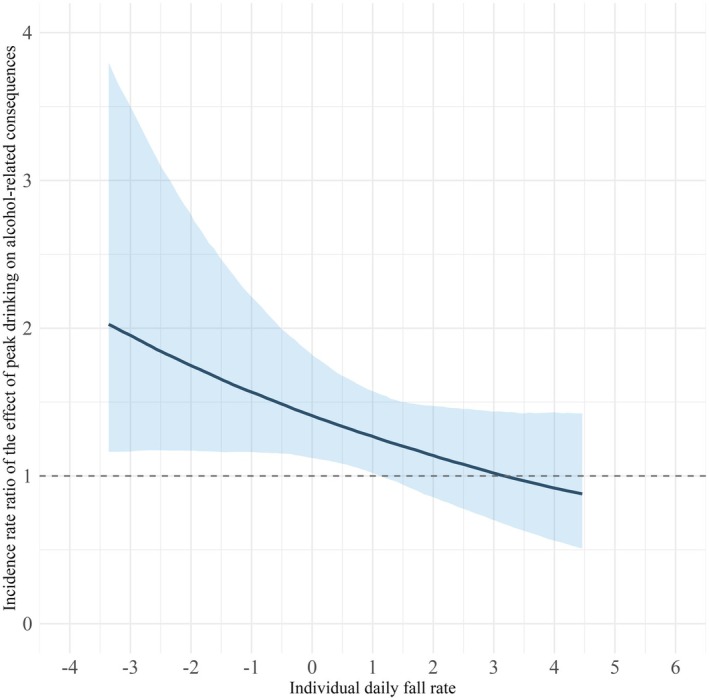
Within‐person association between peak and alcohol‐related consequences differed by daily fall rate (study two). The within‐person association between peak drinking and alcohol‐related consequences varied by daily fall rate, with the person‐mean fall rate held constant. The x‐axis represents individuals' daily fall rates (standard deviations above or below each participant's mean fall rate), and the y‐axis represents the incidence rate ratio (IRR) of the effect of peak drinking on alcohol‐related consequences. The gray shaded area indicates the credible interval.

Similar to Study 1, in Study 2, the association between daily peak and alcohol‐related consequences was significant when the daily fall rate was slower. A significant interaction was observed in the lower range of daily fall rate (from the 1st to 59th percentiles). On days when participants' within‐person fall rate differed from their weekly average (ranging from −3.36 to 1.22 standard deviations around the weekly mean), each one standard deviation increase in daily fall rate was associated with a 10% decrease in the effect of daily peak on the rate of alcohol‐related consequences (IRR = 0.90, 95% CrI: 0.79, 1.02) (Figure 2). However, despite these differential patterns and their similarity to findings from Study 1, the interaction coefficient in Study 2 was not statistically significant.

Sensitivity analysis was repeated using the speed of drinking (rise rate) instead of peak, and found the same pattern of results. The association between rise rate and alcohol‐related consequences was significantly weaker on days with faster fall rates (see Tables S2a and S2b).

## 4 Discussion

The current study used TAC sensors to test the associations between alcohol elimination rate (fall rate) and alcohol‐related consequences across two studies of young adults who frequently engage in heavy drinking. Our study is novel because it is the first to test whether alcohol elimination rate plays a role in alcohol‐related consequences in natural settings, a relationship that has been frequently discussed but rarely examined (Boyd and Corbin 2018; Cederbaum 2012; Russell et al. 2022). Results showed that although fall rates did not have direct associations with alcohol‐related consequences in all studies, they were important moderators of the association between peak intoxication and alcohol‐related consequences at the daily level. On days with faster‐than‐average alcohol elimination, peak TAC is less strongly associated with alcohol‐related consequences. Although the result was only significant in one study, the effect within a specific range was observed in both datasets using two separate alcohol sensing devices. This consistent pattern suggests the presence of an underlying effect. With a larger sample size and more precise measurement instruments, this effect may reach statistical significance. Therefore, future research with larger and more adequately powered samples is needed to strengthen and support these findings.

The first aim of this study was to determine whether peak and fall rate related to the alcohol‐related consequences. Partial support was observed for H1 and H2. Days with higher TAC peaks were positively associated with alcohol‐related consequences. This is consistent with previous studies (Cederbaum 2012; Jones 2019; Richards, Glenn, et al. 2024; Richards, Turrisi, and Russell 2024; Richards et al. 2025; Russell et al. 2022). Building on H1, modeling random slope variation in H2 allows for a more accurate representation of between‐person differences in sensitivity to the effect of peak alcohol levels on alcohol‐related consequences. Findings suggest that interindividual variability is present in the day‐level association between peak TAC and alcohol‐related consequences, with some showing stronger effects and others showing minimal or negligible associations. At an equivalent peak, slower metabolism sustains a longer intoxication duration linked to intensified neurocognitive and behavioral impairment, in turn, elevating the likelihood of alcohol‐related consequences.

The second aim of this study was to determine whether fall rate moderated the association between peak and alcohol‐related consequences. Partial evidence is found in support of H3, proposing that fall rate counteracted the effect of peak on alcohol‐related consequences. The effect of day‐level peak on alcohol‐related consequences diminished as fall rate increased. Alcohol elimination and intoxication are interdependent, overlapping processes. Alcohol metabolism begins upon a change of BAC and initiates the neutralization of alcohol during intoxication. Fluctuations in fall rate reflect underlying variations in the metabolic processing and excretion of alcohol (Cederbaum 2012). The rate of both procedures determines the duration, intensity, and trajectory of BAC. A lower BAC level reduces the immediate intoxicating effects and the risk for alcohol‐related consequences (Hingson et al. 2017; Walsh et al. 2024). Once a drinking episode ends, individuals with faster alcohol elimination rates can clear alcohol from the bloodstream more efficiently, resulting in a steeper BAC decline over a shorter timeframe and consequently shortening the duration of alcohol exposure (Cederbaum 2012). The metabolic kinetics of alcohol elimination demonstrate that higher alcohol metabolism rates driven by liver enzyme activity facilitate more rapid removal of ethanol from circulation (Jones 2019; Zakhari 2006), thereby reducing BAC levels and mitigating alcohol‐related impairments. Individuals with faster biological metabolism may excrete alcohol more efficiently as well, ultimately enhancing alcohol elimination. Although elimination rates accelerate as BAC rises, a ceiling effect occurs at moderate to high BAC levels, beyond which the rate cannot increase further (Cederbaum 2012; Jones 2019; Simic and Tasic 2007). At normal to high BAC, ADH operates at maximum capacity constantly, making the elimination rate independent of concentration (Cederbaum 2012; Jones 2019; Simic and Tasic 2007).

It is important to note that the effect of fall rate on alcohol‐related consequences may be distorted if a large proportion of participants in a study engage in continued low‐level drinking after all ascending portions are complete. Consuming additional drinks slowly or in low doses after ascending does not raise BAC or alter the descending trajectory of the TAC curve, but it can slow the rate of decline and prolong the duration of intoxication. This extended period impedes TAC from falling as rapidly as it would if drinking had ceased. This may contribute to measurement error in elimination rates measured in the natural environment. Future laboratory studies varying the degree to which low‐level drinking occurs during descending periods of a TAC curve could contribute valuable insights.

It is also important to note that the results of this study do not support causal claims that efforts to increase alcohol elimination rates would directly reduce the likelihood of alcohol‐related consequences or mitigate the impact of peak intoxication on such outcomes. Neither the current study nor prior literature provides empirical evidence that alcohol elimination rates can be intentionally modified (Cederbaum 2012; Jones 2019; Russell et al. 2022). Fall rate characterizes the downward trajectory of alcohol concentration. Its variation is contingent on drinking patterns, TAC sensor readings, and peak levels, and may be distinct from the true alcohol elimination rate given that it may, at least partly, represent continued low‐level drinking that fails to increase one's BAC (Richards, Glenn, et al. 2024; Richards, Turrisi, and Russell 2024; Russell et al. 2022). Our results note an association between faster metabolism of alcohol and reduced impacts of high peak intoxication. However, this study does not provide evidence that the drinker can increase their fall rate with the intention of preventing the alcohol related consequences associated with high‐peaked drinking. Recent literature indicates that alcohol metabolism may differ between individuals or vary across days (Cederbaum 2012), but there is no clear evidence that alcohol metabolism can be intentionally accelerated. Additional research is needed to clarify whether a causal relationship exists between alcohol elimination rate and alcohol‐related consequences.

## 5 Limitations

Several limitations in this study should be addressed in future research. First, the findings from the two studies may not generalize to the broader college student population, as the majority of participants were White college students from a single university with a history of heavy drinking. College students who drink infrequently or belong to other racial or ethnic groups may exhibit different relationships between fall rate and alcohol‐related consequences. Second, Study 1 spanned six consecutive days within a single week. Many participants may have engaged in only one drinking event during this period, which limits the ability to analyze within‐person variation in fall rate. Additionally, Study 2 included major holidays. The frequency of intense or binge drinking may have been higher on days like St. Patrick's Day and lower on holidays such as Easter. Such a study period may not accurately reflect typical drinking behaviors among college students if measured over a longer timeline. Fourth, lower‐level drinking behaviors may not have been detected by TAC sensors due to technical limitations. Device‐level issues, such as sensor drift or poor skin contact, can distort the TAC curve and artificially alter the apparent rate of decline. Individual skin properties may influence the diffusion of alcohol through the skin, resulting in variability in the observed decrease in TAC levels. Movement, sensor placement, and device fit may further contribute noise to the signal. Our detection rates are commensurate with previous research (Courtney et al. 2023), but better detection rates must await further advancements in alcohol sensing technology.

## 6 Conclusion

This study provides a foundation for future research exploring how fall rate is linked to alcohol‐related outcomes. Our novel results show that alcohol elimination rate may buffer the impact of peak intoxication on alcohol‐related consequences. These results also contribute to a limited body of research that suggests the rate of alcohol elimination might mitigate some of the negative impacts associated with high peak intoxication. Future research is needed to support these findings and inform health interventions targeting the reduction of alcohol‐related harms.

## Funding

This work was supported by the National Institutes on Drug Abuse, P50DA039838, T32 DA017629, P30CA225520. Oklahoma Tobacco Settlement Endowment Trust, STCST00400_FY25.

## Conflicts of Interest

The authors declare no conflicts of interest.

## Supporting information


**Table S1a:** Descriptive statistics for study variables of Study 1.
**Table S1b:** Descriptive statistics for study variables of Study 2
**Table S2a:** Regression results predicting the alcohol related consequences of Study 1.
**Table S2b:** Regression results predicting the alcohol related consequences of study 2.
**Table S3a:** Distribution of number of drinks on drinking days of Study 1.
**Table S3b:** Distribution of number of drinks on drinking days of Study 2.
**Table S4a:** Alcohol‐related consequences and percentages of Study 1.
**Table S4b:** Alcohol‐related consequences and percentages of Study 2.

## Data Availability

The data that support the findings of this study are available from the corresponding author upon reasonable request.
