# Supplementary material for: The Role of Fall Rate From Transdermal Alcohol Concentration on Alcohol‐Related Consequences in College Students
Source: Alcohol Clin Exp Res (Hoboken). 2026 Jul 9;50(7):e70369. doi: 10.1111/acer.70369 (PMC13347275; doi:10.1111/acer.70369)
Supplement: Supplementary file 1 — Table S1a: Descriptive statistics for study variables of Study 1. Table S1b: Descriptive statistics for study variables of Study 2 Table S2a: Regression results predicting the alcohol related consequences of Study 1. Table S2b: Regression results predicting the alcohol related consequences of study 2. Table S3a: Distribution of number of drinks on drinking days of Study 1. Table S3b: Distribution of number of drinks on drinking days of Study 2. Table S4a: Alcohol‐related consequences and percentages of Study 1. Table S4b: Alcohol‐related consequences and percentages of Study 2. [file ACER-50-0-s001.docx]

| **TABLE 1b: Descriptive statistics for study variables of study 2** | | | | | | | |
| --- | --- | --- | --- | --- | --- | --- | --- |
|  | N persons | N weeks | N days | Mean | Person-level SD | Week-level SD | Day-level SD |
| Alcohol-related consequences | 79 | 247 | 686 | 0.920 | 0.540 | 0.190 | 1.240 |
| Peak | 79 | 247 | 718 | 120.870 | 66.550 | 23.480 | 148.040 |
| Fall rate | 79 | 246 | 717 | 41.1220 | 18.040 | 0.000 | 48.310 |

| **TABLE 1a: Descriptive statistics for study variables of study 1** | | | | | | | | | | | | |
| --- | --- | --- | --- | --- | --- | --- | --- | --- | --- | --- | --- | --- |
|  | | N persons | | N days | | Mean | | Median | | Person-level SD | | Day-level SD |
| Alcohol-related consequences | | 209 | | 553 | | 0.530 | | 0.00 | | 0.430 | | 0.930 |
| Peak | | 218 | | 1274 | | 0.060 | | 0.009 | | 0.040 | | 0.090 |
| Fall rate (- TAC/h) | 218 | | 1270 | | 0.009 | | 0.003 | | 0.005 | | 0.010 | |

| **Table** **2a: Regression results predicting the alcohol related consequences of study 1** | | | |  | |  |
| --- | --- | --- | --- | --- | --- | --- |
| Model 1 | *Fixed Effects* | **IRR** | **CrI_low** | | **CrI_high** | |
|  | Intercept | 0.17 | 0.11 | | 0.25 | |
|  | Daily fall rate | 1.28 | 1.01 | | 1.60 | |
|  | Daily rise rate | 1.32 | 1.02 | | 1.65 | |
|  | Person-mean fall rate | 0.99 | 0.79 | | 1.21 | |
|  | Person-mean rise rate | 1.52 | 1.19 | | 1.98 | |
|  | Female (ref: Male) | 1.15 | 0.83 | | 1.48 | |
|  | Weight | 0.98 | 0.72 | | 1.30 | |
|  | Weekday (ref: Weekend) | 1.21 | 0.96 | | 1.52 | |
|  | *Random Effects* | **Estimates** | **CrI_low** | | **CrI_high** | |
|  | SD of random   intercept | 0.95 | 0.67 | | 1.28 | |
| Model 2 | *Fixed Effects* | **IRR** | **CrI_low** | | **CrI_high** | |
|  | Intercept | 0.14 | 0.08 | | 0.21 | |
|  | Daily fall rate | 1.54 | 1.1 | | 2.08 | |
|  | Daily rise rate | 1.33 | 0.96 | | 1.78 | |
|  | Person-mean fall rate | 0.89 | 0.68 | | 1.12 | |
|  | Person-mean rise rate | 1.2 | 0.85 | | 1.61 | |
|  | Female (ref: Male) | 1.12 | 0.81 | | 1.49 | |
|  | Weight | 0.95 | 0.67 | | 1.26 | |
|  | Weekday (ref: Weekend) | 1.22 | 0.97 | | 1.51 | |
|  | *Random Effects* | **Estimates** | **CrI_low** | | **CrI_high** | |
|  | SD of random intercept | 1.19 | 0.81 | | 1.62 | |
|  | SD of random slope of daily fall rate | 0.43 | 0.00 | | 0.87 | |
|  | SD of random slope of daily rise rate | 0.37 | 0.00 | | 0.83 | |
|  | Correlation between random intercept and random slope of daily fall rate | -0.63 | -0.99 | | 0.07 | |
|  | Correlation between random intercept and random slope of daily rise rate | 0.02 | -0.77 | | 0.80 | |
|  | Correlation between random slope of daily fall rate and random slope of daily rise rate | -0.52 | -0.99 | | 0.57 | |
| Model 3 | *Fixed Effects* | **IRR** | **CrI_low** | | **CrI_high** | |
|  | Intercept | 0.11 | 0.05 | | 0.18 | |
|  | Daily fall rate | 2.31 | 1.46 | | 3.41 | |
|  | Daily rise rate | 1.89 | 1.23 | | 2.69 | |
|  | Person-mean fall rate | 0.91 | 0.61 | | 1.28 | |
|  | Person-mean rise rate | 1.14 | 0.77 | | 1.56 | |
|  | Interaction: Daily rise rate X Daily fall rate | 0.74 | 0.6 | | 0.89 | |
|  | Interaction: Daily rise rate X Person-mean fall rate | 1.02 | 0.85 | | 1.21 | |
|  | Female (ref: Male) | 1.16 | 0.84 | | 1.53 | |
|  | Weight | 0.95 | 0.66 | | 1.26 | |
|  | Weekday (ref: Weekend) | 1.17 | 0.93 | | 1.44 | |
|  | *Random Effects* | **Estimates** | **CrI_low** | | **CrI_high** | |
|  | SD of random intercept | 1.42 | 0.99 | | 1.87 | |
|  | SD of random slope of daily fall rate | 0.68 | 0.31 | | 1.09 | |
|  | SD of random slope of daily rise rate | 0.36 | 0.00 | | 0.79 | |
|  | Correlation between random intercept and random slope of daily fall rate | 0.04 | -0.72 | | 0.83 | |
|  | Correlation between random intercept and random slope of daily rise rate | -0.83 | -1.00 | | -0.49 | |
|  | Correlation between random slope of daily fall rate and random slope of daily rise rate | -0.40 | -0.96 | | 0.57 | |
| * All variables are centered and standardized. Significant fixed effects (CrIs that do not contain 0.0) are bolded | | | | | |  |

| **Table 2b: Regression results predicting the alcohol related consequences of study 2** | | | | | |
| --- | --- | --- | --- | --- | --- |
| Model 1 | *Fixed Effects* | **IRR** | **CrI_low** | **CrI_high** |  |
|  | Intercept | 0.59 | 0.46 | 0.73 |  |
|  | Daily fall rate | 1.41 | 1.09 | 1.80 |  |
|  | Week-mean fall rate | 1.49 | 0.76 | 2.43 |  |
|  | Person-mean fall rate | 0.56 | 0.17 | 1.19 |  |
|  | Daily rise rate | 1.22 | 0.92 | 1.57 |  |
|  | Week-mean rise rate | 0.79 | 0.38 | 1.32 |  |
|  | Person-mean rise rate | 1.55 | 0.40 | 3.61 |  |
|  | Female | 0.81 | 0.63 | 0.99 |  |
|  | Weights | 1.16 | 0.91 | 1.44 |  |
|  | *Random Effects* | **Estimates** | **CrI_low** | **CrI_high** |  |
|  | SD of random intercept | 0.49 | 0.20 | 0.75 |  |
|  | SD of random intercept at the week | 0.24 | 0.00 | 0.57 |  |
| Model 2 | *Fixed Effects* | **IRR** | **CrI_low** | **CrI_high** |  |
|  | Intercept | 0.58 | 0.45 | 0.73 |  |
|  | Daily fall rate | 1.41 | 1.07 | 1.81 |  |
|  | Week-mean fall rate | 1.53 | 0.79 | 2.5 |  |
|  | Person-mean fall rate | 0.56 | 0.17 | 1.2 |  |
|  | Daily rise rate | 1.22 | 0.89 | 1.59 |  |
|  | Week-mean rise rate | 0.78 | 0.38 | 1.32 |  |
|  | Person-mean rise rate | 1.55 | 0.38 | 3.7 |  |
|  | Female | 0.80 | 0.63 | 0.99 |  |
|  | Weights | 1.17 | 0.92 | 1.45 |  |
|  | *Random Effects* | **Estimates** | **CrI_low** | **CrI_high** |  |
|  | SD of random intercept | 0.48 | 0.14 | 0.77 |  |
|  | SD of random slope of fall rate | 0.11 | 0.00 | 0.32 |  |
|  | SD of random slope of peak | 0.13 | 0.00 | 0.39 |  |
|  | SD of random intercept at the week | 0.25 | 0.00 | 0.60 |  |
|  | Correlation between the random intercept and the random slope of daily fall rate | 0.06 | -0.82 | 0.90 |  |
|  | Correlation between the random intercept and the random slope of daily rise rate | -0.15 | -0.94 | 0.75 |  |
|  | Correlation between the random slope of daily fall rate and the random slope of daily rise rate | -0.25 | -0.98 | 0.73 |  |
| Model 3 | *Fixed Effects* | **IRR** | **CrI_low** | **CrI_high** |  |
|  | Intercept | 0.59 | 0.45 | 0.74 |  |
|  | Daily fall rate | 1.38 | 1.05 | 1.72 |  |
|  | Week-mean fall rate | 2.20 | 1.25 | 3.53 |  |
|  | Person-mean fall rate | 0.78 | 0.22 | 1.72 |  |
|  | Daily rise rate | 1.24 | 0.94 | 1.59 |  |
|  | Week-mean rise rate | 0.91 | 0.53 | 1.47 |  |
|  | Person-mean rise rate | 1.85 | 0.51 | 4.17 |  |
|  | Interaction: daily rise rate X daily fall rate | 0.81 | 0.66 | 1.02 |  |
|  | Interaction: daily rise rate X person-mean fall rate | 1.16 | 0.90 | 1.43 |  |
|  | Female | 0.90 | 0.79 | 1.04 |  |
|  | Weights | 1.05 | 0.74 | 1.51 |  |
|  | *Random Effects* | **Estimates** | **CrI_low** | **CrI_high** |  |
|  | SD of random intercept | 0.48 | 0.14 | 0.80 |  |
|  | SD of random slope of fall rate | 0.13 | 0.00 | 0.36 |  |
|  | SD of random slope of rise rate | 0.17 | 0.00 | 0.47 |  |
|  | SD of random intercept at the week | 0.32 | 0.00 | 0.66 |  |
|  | Correlation between the random intercept and the random slope of daily fall rate | 0.06 | -0.76 | 0.93 |  |
|  | Correlation between the random intercept and the random slope of daily rise rate | -0.24 | -0.95 | 0.72 |  |
|  | Correlation between the random slope of daily fall rate and the random slope of daily rise rate | -0.16 | -0.98 | 0.78 |  |
| * All variables are centered and standardized. Significant fixed effects (CrIs that do not contain 0.0) are bolded | | | | |  |

| **Table 3a: distribution of number of drinks on drinking days of study 1** | | |
| --- | --- | --- |
| **Number of drinks per day** | **N** | **%** |
| 0 | 4 | 0.70 |
| 1 | 26 | 4.90 |
| More than 1 | 506 | 94.40 |

| **Table 3b: distribution of number of drinks on drinking days of study 2** | | |
| --- | --- | --- |
| **Number of drinks per day** | **N** | **%** |
| 0 | 35 | 6.20 |
| 1 | 191 | 34.00 |
| More than 1 | 562 | 59.80 |

| **Table 4a. Alcohol-related consequences and percentages of study 1** | | | | | |
| --- | --- | --- | --- | --- | --- |
|  |  |  |  |  |  |
| As a result of drinking yesterday, did you…? | Drinking Days | |  | People | |
|  | N | % |  | N | % |
| have a hangover | 127 | 22.88 |  | 85 | 40.67 |
| feel sick to your stomach | 55 | 9.98 |  | 43 | 20.57 |
| get into an argument with someone | 21 | 3.81 |  | 18 | 8.61 |
| throw up | 19 | 3.45 |  | 19 | 9.09 |
| end up in bad physical “shape” the next day | 18 | 3.24 |  | 17 | 8.13 |
| blackout, or find yourself unable to remember large stretches of time from last night | 16 | 2.90 |  | 13 | 6.22 |
| pass out | 15 | 2.70 |  | 11 | 5.26 |
| do something sexually you wouldn’t have done if you hadn’t been drinking | 11 | 1.98 |  | 9 | 4.31 |
| wake up in an unexpected place | 9 | 1.63 |  | 7 | 3.35 |
| find yourself in a situation where no one was sober enough to drive | 6 | 1.08 |  | 6 | 2.87 |
| have a sexual experience you wish you hadn’t | 3 | 0.54 |  | 3 | 1.44 |
| get in trouble with the police or campus authorities for drinking | 0 | 0.00 |  | 0 | 0.00 |
| get into a physical fight with someone | 0 | 0.00 |  | 0 | 0.00 |

| **Table 4b. Alcohol-related consequences and percentages of study 2** | | | | | |
| --- | --- | --- | --- | --- | --- |
|  |  |  |  |  |  |
| As a result of drinking yesterday, did you…? | Drinking Days | |  | People | |
|  | N | % |  | N | % |
| have a hangover | 200 | 22.88 |  | 85 | 23.88 |
| pass out | 27 | 3.09 |  | 20 | 5.62 |
| get in trouble with the police or penn state authorities for drinking | 3 | 0.34 |  | 3 | 0.84 |
| end up in bad physical shape the next day | 56 | 6.41 |  | 27 | 7.58 |
| find yourself in a situation where no one was sober enough to drive | 38 | 4.35 |  | 21 | 5.90 |
| do something sexually you wouldn’t have done if you hadn’t been drinking | 23 | 2.63 |  | 19 | 5.34 |
| have a sexual experience you wish you hadn’t | 6 | 0.69 |  | 6 | 1.69 |
| wake up in an unexpected place | 11 | 1.26 |  | 9 | 2.53 |
| blackout, or find yourself unable to remember large stretches of time from last night | 49 | 5.61 |  | 32 | 8.99 |
| throw up | 37 | 4.23 |  | 32 | 8.99 |
| feel sick to your stomach | 130 | 14.87 |  | 62 | 17.42 |
| get into a physical fight with someone | 3 | 0.34 |  | 3 | 0.84 |
| get into an argument with someone | 58 | 6.40 |  | 37 | 10.39 |
